# Fisheries governance in the face of climate change: Assessment of policy reform implications for Mexican fisheries

**DOI:** 10.1371/journal.pone.0222317

**Published:** 2019-10-02

**Authors:** Miguel Angel Cisneros-Mata, Tracey Mangin, Jennifer Bone, Laura Rodriguez, Sarah Lindley Smith, Steven D. Gaines

**Affiliations:** 1 Instituto Nacional de Pesca y Acuacultura, Guaymas, Sonora, Mexico; 2 Bren School of Environmental Science & Management, University of California Santa Barbara, Santa Barbara CA, United States of America; 3 Sustainable Fisheries Group, Bren School of Environmental Science & Management, University of California Santa Barbara, Santa Barbara, CA, United States of America; 4 Environmental Defense Fund de México A.C., La Paz, BCS, México; 5 Environmental Defense Fund, Boston, MA, United States of America; Swedish University of Agricultural Sciences and Swedish Institute for the Marine Environment, University of Gothenburg, SWEDEN

## Abstract

Climate change is driving shifts in the abundance and distribution of marine fish and invertebrates and is having direct and indirect impacts on seafood catches and fishing communities, exacerbating the already negative effects of unsustainably high fishing pressure that exist for some stocks. Although the majority of fisheries in the world are managed at the national or local scale, most existing approaches to assessing climate impacts on fisheries have been developed on a global scale. It is often difficult to translate from the global to regional and local settings because of limited relevant data. To address the need for fisheries management entities to identify those fisheries with the greatest potential for climate change impacts, we present an approach for estimating expected climate change-driven impacts on the productivity and spatial range of fisheries at the regional scale in a data-poor context. We use a set of representative Mexican fisheries as test cases. To assess the implications of climate impacts, we compare biomass, harvest, and profit outcomes from a bioeconomic model under contrasting management policies and with and without climate change. Overall results show that climate change is estimated to negatively affect nearly every fishery in our study. However, the results indicate that overfishing is a greater threat than climate change for these fisheries, hence fixing current management challenges has a greater upside than the projected future costs of moderate levels of climate change. Additionally, this study provides meaningful first approximations of potential effects of both climate change and management reform in Mexican fisheries. Using the climate impact estimations and model outputs, we identify high priority stocks, fleets, and regions for policy reform in Mexico in the face of climate change. This approach can be applied in other data-poor circumstances to focus future research and policy reform efforts on stocks now subject to additional stress due to climate change. Considering their growing relevance as a critical source of protein and micronutrients to nourish our growing population, it is urgent for regions to develop sound fishery management policies in the short-term as they are the most important intervention to mitigate the adverse effects of climate change on marine fisheries.

## Introduction

By altering marine habitats and oceanographic conditions, climate change is having significant impacts on marine fisheries around the globe, affecting the distribution and productivity of numerous marine fish and invertebrate stocks and creating a source of uncertainty and risk for fishing industries, coastal communities, and the millions of fishers whose livelihoods and food security depend on fisheries [1–4]. Further, for many nations, climate change has the potential to exacerbate the negative effects of unsustainably high levels of fishing pressure on stocks, affecting profitability for both industrial and small-scale fishing fleets [5–11].

Climate change affects physical conditions (e.g., sea surface temperature, acidity, salinity, and oxygen levels) of the ocean environment [7,12], which subsequently affects marine species by altering biogeochemical cycles, trophic flows, as well as species life histories, productivity, and distributions [13–18]. It is well documented that marine populations have spatially shifted in response to increases in ocean temperature [12,13,16,19]. These shifts may further jeopardize vital food sources and livelihoods for people who rely on fisheries, particularly in regions where declines from overfishing are already occurring. Stocks are projected to decline in productivity, and it is predicted that species will spatially shift poleward and deeper where they may become inaccessible to those fishers who have fished for them historically [7,14,20–22].

Global estimates of climate change effects on fishery biomass, harvest, and profits, while useful for understanding the broad implications of climate change and management interventions, may be insufficient for guiding policy and management decisions at the scales where governance institutions operate (typically country level). Gaines et al. find that although global fishery catches and profits can be greater in the future compared to today under moderate climate change, outcomes vary dramatically among different fisheries and regions, suggesting that fishery potential and the appropriate interventions will differ across the globe [17]. Moreover, simplifications that allow reasonable global estimates of change (e.g., dynamics driven by climate velocity, estimates at the species-level as opposed to the stock-level [17,21]) are unlikely to provide the level of accuracy needed for regional and local decision-making in the face of a broader range of potential climate change threats. More comprehensive analyses that are at appropriate socio-economic and governance scales and incorporate more localized climate effects that are not captured in global models will improve predictions and enable managers to more aptly respond to climate threats [5,8,23]. Additionally, regional climate change predictions are particularly challenging within data-poor contexts, where there is limited information available regarding stock status and how climate change will impact fishery productivity and spatially shift stocks’ ranges. Unassessed small-scale fisheries are estimated to be in worse condition than those that are assessed, and may be more vulnerable to climate change effects due to limited governance capacity [8,24].

Mexico provides a useful case study for a globally significant fishing nation with limited available information on current fisheries status and anticipated climate change effects on fisheries. In 2016, Mexico ranked 16^th^ in global marine capture production with landings totaling 1.31 million metric tons (MT) [25]. A recent study found that out of 735 species caught in 83 fisheries, the majority are unassessed or assessments are not publicly available [26]. Of the stocks that have a declared status for year 2018 in Mexico’s National Fisheries Chart, 14.3% are overexploited, 80% are being exploited at their maximum level and just 5.7% have potential for expansion [26]. Inadequate and ineffective fisheries management [27] coupled with climate change impacts threaten not only Mexico’s emerging role as a global exporter of fisheries products [25], but also the nearly 240 thousand fishers who rely directly on these resources for their livelihoods [25].

### Aims

The aim of this study is to develop a replicable approach to estimate–at a regional scale and under a data-poor context–the implications of climate change and management on fishery biomass, harvest, and profit. To achieve this, we first develop an approach to estimate the magnitude and direction of change associated with climate effects on a fishery. Due to the data limitations, our aim is not to make accurate estimates but instead to provide initial estimates that can inform further analysis and management prioritization. We then use a model to determine the implication of these effects on fishery biomass, harvest, and profit under different management scenarios. This work builds on previous efforts in two ways: 1) it offers a procedure for parameterizing climate change models based on scarce information, and 2) it focuses on regional distributional effects of climate impacts on fishery resources, which provide insights at a scale aligned with that of governance and socio-economic institutions. We believe that this method offers a useful tool for fishery researchers in other regions where data-poor conditions necessitate new approaches that provide general trends and inform further analytical priorities.

Using this subset of Mexican fisheries, we explore the following questions on a national scale:

1. Based on available information and input from experts on climate change effects on fish populations, how will climate change affect the productivity and accessibility of a representative set of Mexico’s fisheries?
2. How do different fisheries perform in terms of biomass, catch, and profit under a combination of both different management scenarios and under the anticipated impacts of climate change?
3. Can improved management lead to better outcomes under climate change compared to status quo management without climate change?
4. What are the distributional effects of climate change on different fleets in our study?

## Materials and methods

We develop and employ an approach to forecast local climate change effects on fisheries by linking estimates of current fisheries status and information regarding expected regional climate effects with a bioeconomic model that projects future biomass, catch, and profits under alternative scenarios, using a set of Mexican fisheries as case studies. We compare the distributional effects of climate change on artisanal (i.e., small-scale) and industrial (i.e., large-scale) fisheries, as the latter can generally adapt better to spatial range shifts (e.g., poleward shifts, vertical migrations to deeper waters) than the former.

Our study analyzed 25 fished stocks, which together account for over 70% of Mexico’s total landings in 2012 and are representative of different climatic regions and ecological environments. The stocks considered in the present study include four stocks targeted by industrial fisheries using large vessels with automated equipment, sixteen stocks targeted by artisanal fisheries from small (<11 m length) boats or pangas, and five stocks shared by industrial and artisanal fleets (referred to as “mixed”) (Table 1). All fisheries are managed nationally, with the exception of yellowfin tuna, which is managed by international management bodies. Although the yellowfin tuna fishery experiences foreign fishing pressure, we do not separate this from domestic fishing pressure in order to preserve stock dynamics.

**Table 1:** Mexican fished stocks included in this study. Fishing range (in nautical miles, nm) represents distances traveled by vessels to harvest their target resources.

Common Name (English)	Common Name (Spanish)	Region	Scientific Name	Fleet type	Gear(s)	Fishing range
Black murex snail	Caracol chino negro	Sonora	*Hexaplex nigritus*	Artisanal	Free and hookah diving, pots, gillnets	1 to 40 nm
Brown swimming crab	Jaiba café	Sonora and Sinaloa	*Callinectes bellicosus*	Artisanal	Pots, gillnets	1 to 40 nm
Cannonball jellyfish	Medusa bola de cañón	Gulf of California	*Stomolophus* spp.	Artisanal	Hand scoops	0.5 to 30 nm
Chocolate clam	Almeja chocolata	Baja California Sur	*Megapteria squalida*	Artisanal	Free and hookah diving	1 to 50 nm
Geoduck	Almeja generosa	Upper Gulf of California	*Panopea globosa*	Artisanal	Hookah diving	1 to 30 nm
Gulf corvina	Curvina golfina	Upper Gulf of California	*Cynoscion othonopterus*	Artisanal	Small purse seine	1 to 40 nm
Lion-paw clam	Almeja mano de león	Baja California Sur	*Lyropecten subnodosus*	Artisanal	Hookah diving	1 to 50 nm
Pacific abalone	Abulón azul	Mexican North Pacific	*Haliotis fulgens*	Artisanal	Hookah diving	1 to 40 nm
Penshell scallop	Callo de hacha	Bahía de Kino, Sonora	*Atrina tuberculosa*	Artisanal	Hookah diving	1 to 30 nm
Queen conch	Caracol rosado	Yucatán Peninsula	*Strombus gigas*	Artisanal	Free and hookah diving	1 to 40 nm
Red snapper	Huachinango	Gulf of California	*Lutjanus peru*	Artisanal	Hand lines, gill nets, long lines	1 to 30 nm
Sea cucumber	Pepino de mar	Gulf of California	*Isostichopus fuscus*	Artisanal	Hookah diving, free diving	1 to 40 nm
Snook	Robalo	Sinaloa	*Centropomus robalito*	Artisanal	Gill nets	1 to 30 nm
Spanish mackerel	Sierra	Sonora	*Scomberomorus* spp.	Artisanal	Mostly gill nets	1 to 40 nm
Spiny lobster	Langosta	Mexican North Pacific	*Panulirus interruptus*	Artisanal	Pots	1 to 40 nm
Triggerfish	Pez cochito	Sonora	*Ballistes polylepis*	Artisanal	Pots, gillnets, hand lines	1 to 40 nm
Pacific hake	Merluza	Northern Gulf of California	*Merluccius productus*	Industrial	Trawlers	20 to 300 nm
Pacific sardine	Sardina Monterrey	Gulf of California	*Sardinops sagax*	Industrial	Purse seiners	1 to > 300 nm
Pelagic red crab	Langostilla	Baja California Sur	*Pleuroncodes planipes*	Industrial	Trawlers	10 to 100 nm
Yellowfin tuna	Atún aleta amarilla	Mexican Pacific	*Thunnus albacares*	Industrial	Purse seiners	1 to > 300 nm
Black tip shark	Tiburón de puntas negras	Gulf of Mexico	*Carcharhinus limbatus*	Mixed	Hand lines, gill nets, long lines	1 to 50 nm
Blue shrimp	Camarón azul	Gulf of California	*Litopenaeus stylirostris*	Mixed	Industrial: trawlers; Artisanal: cast nets, gill nets, small trawlers	Industrial: 1 to > 300 nm; artisanal: 5 to 20 nm
Jumbo squid	Calamar gigante	Gulf of California	*Dosidicus gigas*	Mixed	Industrial: automatized jigs; Artisanal: mostly hand jigs	Industrial: > 100 nm; artisanal: 1 to 40 nm
Mahi-mahi	Dorado	Mexican Pacific	*Coryphaena* spp.	Mixed	Artisanal: gill nets, trolls; industrial: purse seine	Artisanal: 5 to 40 nm; industrial: 50 to > 300 nm
Red grouper	Mero	Campeche Bank	*Epinephelus morio*	Mixed	Artisanal and industrial: hand lines, long lines	1 to 120 nm

### Model description

To forecast regional climate change effects on fisheries, we use a bioeconomic model based on Costello et al. [27], which pairs a Pella-Tomlinson’s surplus production model [28] with an economic model to project future biomass, harvest, and profit over a thirty-year time horizon.

The Pella-Tomlinson model [28] is a generalized version of the logistic growth model and provides biological time-dynamics for each fishery. We chose this model for its flexibility (the Fox and Schaefer models are special cases of the Pella-Tomlinson) [29]. The model is given as follows:

$$B_{t+1}=B_{t}+\frac{\phi +1}{\phi }g_{t}B_{t}\left(1-{\left(\frac{B_{t}}{K_{t}}\right)}^{\phi }\right)-H_{t}$$ (1)

where, for each year *t*, *B*_*t*_ is biomass, *g*_*t*_ is the growth parameter, *K*_*t*_ is carrying capacity, *H*_*t*_ is annual harvest, and *ϕ* is Pella-Tomlinson’s shape parameter. The economic model is:

$$\pi_{t}=pH_{t}-c{\left(F_{t}\right)}^{\beta }$$ (2)

where *π*_*t*_ is profit (revenues minus costs) in year *t*, *p* is the ex-vessel fixed price, *F*_*t*_ is the fishing mortality rate, *c* is a variable cost parameter, and *β* governs the shape of the cost per unit effort. Harvest is calculated as follows:

$$H_{t}=F_{t}B_{t}$$ (3)

Climate change is incorporated by allowing a population’s carrying capacity (*K*) and growth rate (*g*) to change over time. The magnitude and direction of changes are estimated using a parameterization process that incorporates available information and expert opinion (see the *Data and climate parameterization* section for more details). We project future biomass, harvest, and profit trajectories for each fishery over a 30-year time horizon under different climate and management scenarios.

### Data and climate parameterization

We use fishery-specific data and parameters originally developed by Mangin et al. [30] (see S1 Table for parameter values) to parameterize the current fishery status in this study. Biological parameters were determined using information for the species considered when available or for similar species from other regions when local data were unavailable. Economic parameters were developed using official catch statistics and first-hand price information, as well as estimated cost data [31]. Detailed descriptions of criteria, data, and information used in assigning values to starting biological and economic parameters are provided in Mangin et al. [30] and Cisneros-Mata [31].

Climate change effects are parameterized using an approach that determines anticipated long-term relative changes in regionally available biomass because of range shifts (incorporated in the model by allowing carrying capacity *K* to change) and the growth parameter *g*. Functionally, allowing carrying capacity to change affects the maximum amount of potential catch available to a fleet. A major assumption in the present work is that fishers will perceive poleward spatial shifts and vertical migrations as a reduction in available fishable biomass or potential catch. Our rationale is not implying that socioeconomic factors affect *K*; rather, that climate change will affect *in situ* abundance or perceived *K* for fleets. We assume that changes in *K* and *g* occur at a constant rate over time.

Based on the information summarized in Table 2 regarding expected climate change impacts on fish and invertebrates in Mexico, we identified environmental drivers of range shifts and changes in population growth rate. In addition, we included socioeconomic drivers that can influence adaptability to spatial shifts. Environmental factors that impact growth (*g*) are temperature rise, acidification, disease outbreaks, sea level rise, and freshwater inflow. Environmental factors that influence range shifts (*K*) are sea level rise, freshwater inflow, and migration. Catchability and governance are socioeconomic factors that influence adaptive capacity to range shifts.

**Table 2:** Expected and observed impacts of climate change in marine ecosystems, marine fish and invertebrates, and fisheries in Mexico.

Forcing mechanism	Effects	Results	Source
Temperature rise	Poleward/deeper waters shifts	Less in situ fishery biomass	[7]
		Less local catch	
Temperature rise; low upwelling	Nutrient reduction	Less primary productivity	[32]
Temperature rise; low rainfall	Low phytoplankton due to low riverine input	Less biomass	
Temperature rise; decreased upwelling, fishing	Low phytoplankton due to low riverine input	Less local catch	
Temperature rise, salinity, upwelling	Poleward shift	Less local catch	[22]
Temperature rise/decrease; increase/decreased upwelling	Increased turbulence and reduced plankton	Reduced catches	[33]
Temperature and sea level rise	Acidification	Less coral reefs	[34]
Temperature rise	Not specified	Not specified	[35]
Ocean CO_2_ sequestration	Acidification	Echinoderms: low biomass	[36]
		Mollusks: low biomass	
		Crustaceans: low biomass	
		Fish: possible effects on larval survival	
Temperature rise, fishing mortality	Decreased plankton	Mid pelagic fish: increased biomass	[37,38]
		Small pelagics: less biomass	
		Cephalopods: less biomass	
		Bivalves: less biomass	
Temperature, salinity, windfields, oxygen, acidification	Poleward shift, change in productivity and trophic structure of communities	Not specified	[6,22]
Upwelling, temperature	Changes in fished stock productivity	Changes in catchability, particularly short-lived species	[22]
Wind, upwelling, salinity	Effects on plankton and fish dynamics	Change in biomass of small fishes	[39]
		Change in biomass of top predatory fish	
Temperature, rainfall	Habitat loss. Direct: temperature for species with limited thermal ranges. Indirect: poleward movement, habitat changes and more diseases	Urchins: habitat expansion, increased biomass	[40]
		Lobsters: low biomass (affected by urchins)	
		Abalones: low biomass	
		Organic reef species: unchanged	
		Prawns and crabs: increased biomass	
N/S	Poleward shifts	Slight decrease in Mexican marine catches	[5]
Temperature, CO_2_ sequestration	Poleward shifts, acidification, ecosystem disruptions, changes in primary productivity	Less biomass, hence overfishing	[22]
Wind stress	Deep euphotic zone and increased offshore transport	High primary productivity, variable phenology	[41]

For each environmental driver, we assign a value that represents the expected directional (positive, negative, or neutral) effect of climate change and magnitude (none, low, medium, and high) of that impact on the model parameters based on the expected fishery effects found in the literature review (Table 3). The numerical values assigned to each magnitude of change are a 0%, 5%, 10% and 15% in current *g* or *K* for no, low, medium, and high impacts, respectively. We chose these values because more definitive information on the functional relationship between climate change drivers and population parameters *g* and *K* for these taxa is unavailable. Therefore, we instead assigned values intended to represent an increasing magnitude of change in the absence of better empirical data, and chose values that are conservative. Using a process similar to that of Hare et al. [42], we qualitatively assessed impacts for each of the environmental factors for all 25 stocks based on current general knowledge of how climate change will influence species distribution, biology, and physiology (cf., Table 2). We also took into consideration biological and ecological characteristics of stocks included in this study such as geographical location, longevity, fecundity, and habitat temperature.

**Table 3:** Parameterization of environmental and socioeconomic factors that affect productivity and range shifts.

		Environmental factors that affect productivity					Environmental factors that affect productivity and range shifts		Environmental factor that affects range shifts	Socioeconomic factors that affect adaptability to range shifts		Total effect on model parameters (%)	
Stock	Fleet type	Temperature rise		Acidification		Disease outbreaks	Sea level rise	Freshwater inflow	Migration	Catchability	Governance	*g*	*K*
**Black murex snail**	Artisanal	0	-0.05		0		0.05	0	0	0	-0.05	0%	0%
**Brown swimming crab**	Artisanal	0	-0.05		-0.05		0.05	0.05	-0.05	0	-0.05	0%	0%
**Cannonball jellyfish**	Artisanal	0.1	-0.05		0		0.05	0	-0.05	0	-0.05	+10%	-5%
**Chocolate clam**	Artisanal	0	-0.05		0		0.05	-0.05	0	0	-0.05	-5%	-5%
**Geoduck**	Artisanal	0.05	-0.05		0		0.05	-0.03	0	0	-0.05	+2%	-3%
**Gulf corvina**	Artisanal	0	-0.05		0		0.05	-0.05	0	0	0	-5%	0%
**Lion-paw clam**	Artisanal	0	-0.1		0		0.05	0	0	0	-0.05	-6%	0%
**Pacific abalone**	Artisanal	-0.1	-0.1		-0.15		0	-0.05	-0.1	0	0	-35%	-15%
**Penshell scallop**	Artisanal	0	-0.05		0		0.03	0	0	0	-0.1	-3%	-8%
**Queen conch**	Artisanal	0	-0.05		0		0.03	0	0	0	-0.05	-3%	-3%
**Red snapper**	Artisanal	-0.05	-0.05		0		0.03	0	-0.1	-0.05	-0.1	-7%	-21%
**Sea cucumber**	Artisanal	0.05	-0.05		0		0.03	0	0	0	-0.1	+2%	-8%
**Snook**	Artisanal	0	-0.05		0		0.05	0.05	-0.1	0	-0.1	+5%	-11%
**Spanish mackerel**	Artisanal	0	-0.05		0		0	0	-0.05	0	-0.1	-5%	-15%
**Spiny lobster**	Artisanal	0	-0.05		0		0.03	0	-0.1	0	0	-3%	-8%
**Triggerfish**	Artisanal	0	-0.05		0		0	0	0	0	-0.1	-5%	0%
**Pacific hake**	Industrial	0	-0.05		0		0	0	0	0	-0.05	-5%	0%
**Pacific sardine**	Industrial	-0.1	-0.05		0		0	0	-0.15	-0.1	0	-15%	-24%
**Pelagic red crab**	Industrial	-0.1	-0.1		0		0	0	-0.1	-0.1	0	-19%	-19%
**Yellowfin tuna**	Industrial	0	-0.05		0		0	0	-0.05	0	0	-5%	-5%
**Black tip shark**	Mixed	0	0		0		0	0	-0.05	-0.1	-0.1	0	-23%
**Blue shrimp**	Mixed	0	-0.05		-0.05		0	0.03	0	0	-0.05	-7%	-3%
**Jumbo squid**	Mixed	-0.05	-0.05		0		0	0	-0.15	-0.05	-0.05	-10%	-23%
**Mahi-mahi**	Mixed	0	-0.05		0		0	0	-0.1	-0.1	-0.1	-5%	-27%
**Red grouper**	Mixed	-0.05	-0.05		0		0	0	-0.05	0	-0.05	-10%	-10%

As with environmental factors, we assigned values for the two socioeconomic factors, which reflect how fishery innovation and governance can impact the ability of fishers, firms, or industries to adapt to climate driven spatial range shifts. Changes are classified in terms of ability to adapt: no, low, medium, and high abilities to adapt represent -15%, -10%, -5%, and 0% change in current *K*. Fisheries with a low ability to adapt to spatial shifts experience losses in potential catch, while potential catch is unaffected by spatial range shifts for those with a high ability to adapt. We use these values as rough approximations of expected outcomes. We assume that strong governance (e.g., existing and flexible legal, regulatory and management frameworks) and the differing ability of fleets to follow migrations (e.g., fleets that can travel long distances, are more technologically advanced, have greater access to technological advances and capital) affect the ability to adapt to range shifts. Generally, industrial fleets are more mobile and can more easily adapt to range shifts compared to artisanal fishers, who typically have a smaller fishing range because of vessel and gear limitations, and may be more strongly attached to a particular fishing community [43]. Explanations for the values that we chose for each species are provided in S1 Text.

Using the parameterization documented in Table 3, we estimated the total relative impact on productivity (*g*) and range shifts (represented in the model as a change in *K*) for each fishery. We calculated the total relative climate effect on parameters *g* and *K* for each fishery using the following equation:

$$r_{p,f}=\Pi_{f}(1+c_{i,f})-1$$ (4)

where *r* is the relative effect for parameter *p*, *c*_*i*,*f*_ is the relative change for each factor *f* and each stock *i* (see Table 3). For stocks that are not expected to be impacted by sea level rise, species migration, or freshwater inflow, we set the relative impact on *K* = 0, regardless of the parameterization for socioeconomic factors. For factors that are expected to affect both population parameters, we divide the magnitude of the impact evenly between the two parameters. This was done because of a lack of understanding of how forcing mechanisms simultaneously impact both *g* and *K* for the 25 stocks considered in our study. To determine the *g* or *K* values at the end of the time horizon (*g*′ and *K*′), we simply apply the percent change to the original values (*g*_0_ and *K*_0_).

### Management scenarios

We examine future fisheries performance under two management scenarios and two environmental conditions (under current environmental conditions and expected climate change impacts). This results in four hypothetical management and climate change scenarios: 1) status quo management without climate change (SQ no CC), 2) status quo management with climate change (SQ with CC), 3) management to achieve optimal economic output without climate change (Opt no CC), and 4) management to achieve optimal economic output with climate change (Opt with CC).

The status quo (SQ) management policy simply maintains the current fishing mortality rate for each fishery (*F*_*SQ*_ = *F*_0_). The management policy that achieves optimal economic output (Opt) results in the greatest net-present value of profits (i.e., the sum of all discounted future profits) under current climatic conditions. This policy is determined using a dynamic optimization routine for each stock. Unlike the SQ management scenario, the fishing mortality rate in the Opt scenario is not constant, but a function of how much biomass is in the water. This policy enables a reduction in fishing effort when the stock is depleted, and an increase in fishing effort when the stock is healthy.

## Results

### Analysis of climate effects

We estimated long-term changes in productivity and catch potential due to climate change for the 25 stocks (Table 3). For productivity, expected changes range from negative to positive. Pacific abalone is expected to have the largest negative relative change (-35%), cannonball jellyfish is expected to have the largest positive change (10%), and several species, including black tip sharks, black murex snail, and brown swimming crab are expected to experience little change (i.e., < 1% change).

We find that most fisheries [19] in our study will experience reductions in catch potential (> 1% decrease) due to a limited ability to adapt to climate driven spatial range shifts (Table 3). Mahi-mahi is expected to have the largest catch reductions (-27%) followed by Pacific sardine (-24%), jumbo squid and black tip shark (-23%), and red snapper (-21%). The six fisheries that are not expected to experience a reduction in potential catch due to climate driven shifts are triggerfish, Pacific hake, lion-paw clam, black murex snail, Gulf corvina, and brown swimming crab.

**Fig 1:**
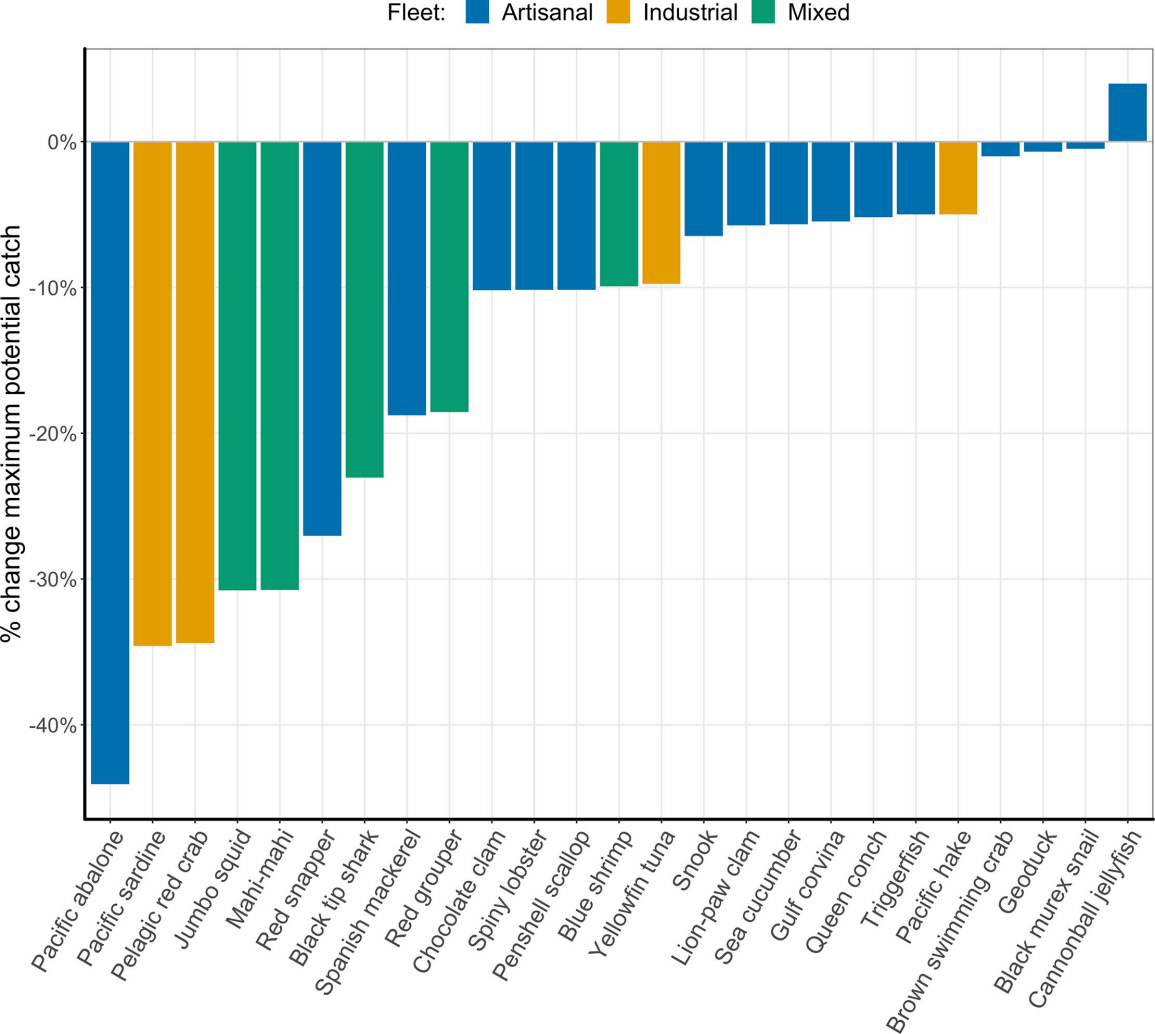
Change in maximum potential catch due to climate change effects: initial MSY compared to maximum potential catch at the end of the thirty-year time horizon. Maximum potential catch for brown swimming crab, geoduck, and black murex snail (all of which are fished by artisanal fleets) are the least affected by climate change. The artisanal fleet is the only fleet with a stock that is positively affected by climate change.

We calculated maximum potential catch at the end of the time horizon using *g* and *K* values adjusted according to Table 3 and the equation in S2 Text from Costello et al. [27]. Maximum potential catch at the end of the thirty-year time horizon is on average 14% lower than initial maximum sustainable yield (MSY) for the stocks in our study (Fig 1). Pacific abalone experiences the greatest declines (-44%), while cannonball jellyfish is the only stocks that experiences an increase (4%). The majority of stocks (84%) experience declines in maximum catch potential greater than 1% over the time horizon.

### Model results

#### Comparing effects of climate change by fleet

**Fig 2:**
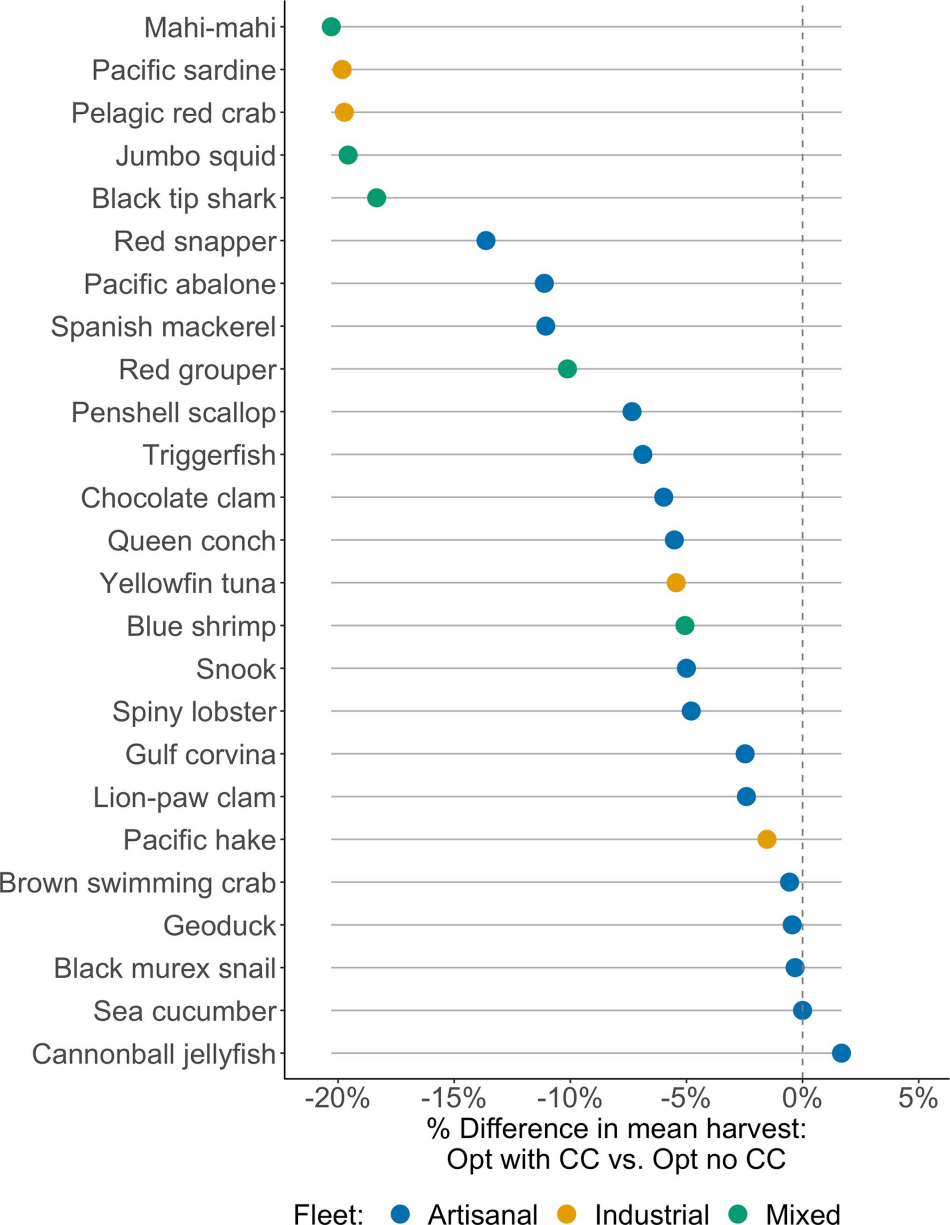
Impact of climate change on mean catch under Opt management. This comparison isolates the climate effect on Mexico’s stocks and shows that for the majority of stocks, mean harvest is expected to be lower under a future with climate change.

To determine the distributional effects of climate change, we compare catch outcomes using the Opt management policy with climate change to the outcomes of using the same policy without climate change for the three fleet groups. The effects on mean annual harvest varies among fisheries within each fleet group. The difference in mean annual harvest ranges from -13.6 to 1.7% for fisheries caught by the artisanal fleet only, -20.3 to -5.1% for those caught by a mix of artisanal and industrial fishers, and -19.8 to -1.5% for the fisheries caught by only the industrial fleet (Fig 2).

#### Effect of management scenarios and climate change

**Fig 3:**
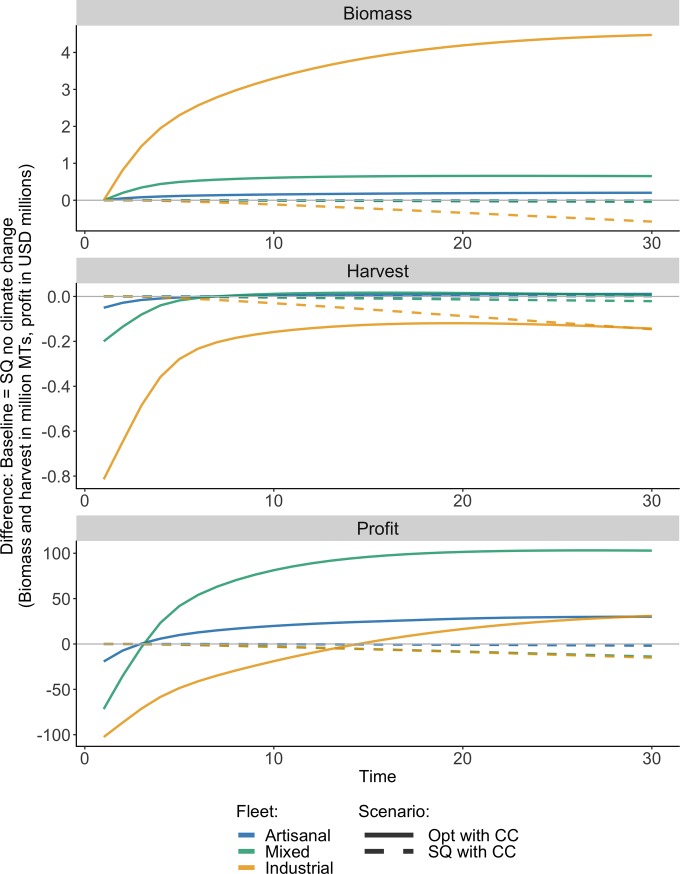
Comparison of fishery outcomes for each fleet. Annual biomass, harvest, and profit indicators for three contrasting scenarios relative to the SQ no CC scenario. For each fleet, the Opt policy results in greater profit compared to the SQ policy (with or without climate change) by year 15.

Results show that, while keeping management the same (SQ), the total annual biomass and profit for all three fleet groups (artisanal, industrial, and mixed) is lower with climate change compared to no climate change (Fig 3). Furthermore, the SQ with CC scenario also leads to lower total annual harvest for the industrial and mixed fleet groups. The fisheries caught by the mixed fleet group experience the greatest relative impact from climate change, with mean biomass and harvest indicators 9.0% and 11.3% lower than those expected with the same policy without climate change. The industrial fleet experiences the greatest loss to mean total profit under climate change, which is 6.5 million USD lower than the mean total profit without climate change.

By contrast, the Opt management policy under a future with climate change leads to higher total annual biomass and profit for all three fleet groups compared to the SQ policy, both with and without climate change (Fig 3). For two of the three fleets (artisanal and mixed), this is achieved while also ultimately reaching annual harvest levels that are higher than those obtained using the SQ policy, again with or without climate change. Mean annual profit is substantially higher for both the artisanal and mixed fleets compared to the SQ no CC scenario (20.5 and 76.1 million USD respectively). Although mean profit for the industrial fleet is lower than that of this baseline scenario, annual profits under the Opt harvest policy with climate change eventually surpass those under the SQ no CC scenario.

**Fig 4:**
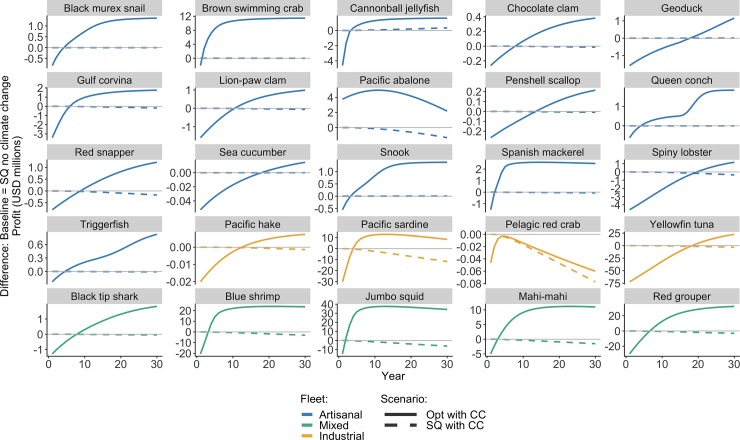
Comparison of profit outcomes for each stock. Annual profit indicators for two contrasting scenarios relative to the SQ no CC scenario. The Opt policy with climate change results in greater profit compared to the SQ policy (with or without climate change) for all stocks except pelagic red crab, which is one of the only stocks in our study considered to be underfished and experiencing overfishing (S1 Table), and is also one of the most vulnerable to climate change (Fig 1).

For most individual fisheries, the Opt policy leads to greater profit than the SQ policy regardless of whether or not climate change occurs, with the exception of Pelagic red crab, for which the Opt with CC scenario leads to greater profit than SQ with CC, but not SQ no CC) (Fig 4). Pelagic red crab is one of three stocks in this study considered to be underfished (B/B_MSY_ > 1) and experiencing underfishing (F/F_MSY_ < 1) (S1 Table), and is the second most vulnerable to climate effects according to the parameterization in Table 3. Therefore, it is not in need of rebuilding and economic benefits of reform would thus be comparatively small. However, this stock is expected to be greatly affected by climate change.

**Fig 5:**
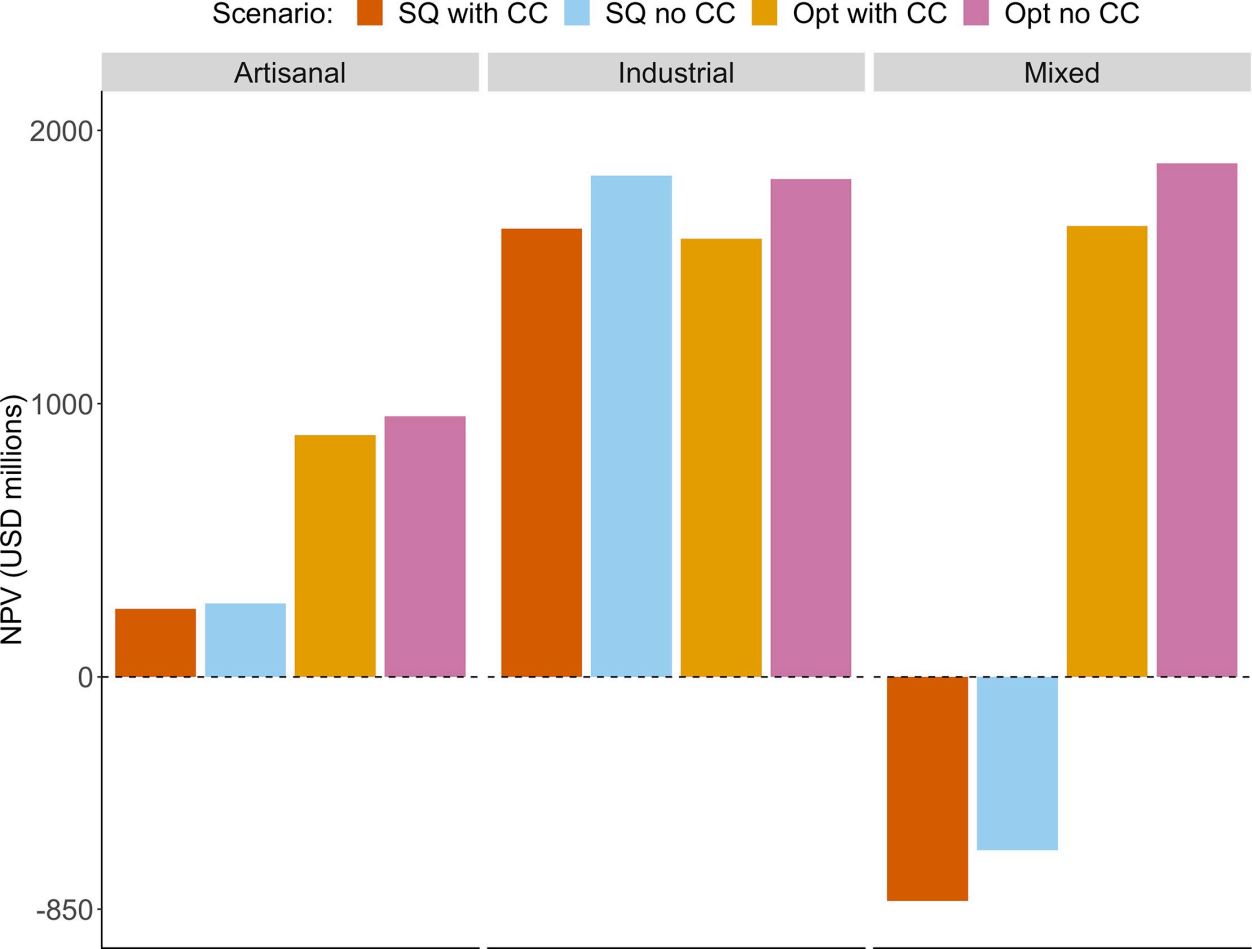
Comparison of total profit for length of projection for the four policy-climate change scenarios. Negative values reflect negative profitability.

When comparing economic outcomes of climate change-policy scenarios to those of Opt no CC, climate change always results in lower net present value (NPV) values, which represents the sum of all annual profits over the course of the projection, regardless of the management option (Fig 5). However, even though climate change leads to losses in NPV, the application of the Opt harvest policy prevents much more severe NPV losses compared to the SQ with CC policy for the artisanal and mixed fleets. Although NPV is higher for the industrial fleet under the SQ management policy, annual trajectories in Fig 4 suggest that this is largely driven by the yellowfin tuna fishery, which becomes more profitable under the Opt policy than the SQ policy in year 19 (Fig 3). Therefore, NPV under the Opt management scenario would eventually surpass that of the SQ scenario (with or without climate change).

## Discussion

Our study finds that most of the stocks analyzed will be negatively impacted by climate change, but that management reforms can mitigate or counteract many of these effects. Based on our parameterization method, climate change will negatively affect potential catch for 84% of fisheries in our study (Fig 1). Only four stocks (cannonball jellyfish, snook, sea cucumber, and geoduck) are expected to experience increases in productivity from climate change; however, range shifts are expected to decrease maximum catch potential for these three stocks. These results align with other estimates of climate effects on fisheries in tropical latitudes and Mexico especially, where climate change is expected to increase vulnerability of fisheries due to increased sea surface temperature, precipitation, sea level, and freshwater runoff [44]. Changes in catch potential due to range shifts have already been observed in the Pacific sardine fishery, which has shifted vertically to deep waters and outside the Gulf of California, and for jumbo squid, which has emigrated from the Gulf of California to coasts in the United States’ Pacific northwest, affecting industrial and artisanal fishers, respectively [45,46]. Stocks that are estimated to be particularly vulnerable to productivity declines (10% or greater estimated decline in *g*) are Pacific abalone, pelagic red crab, and Pacific sardine (the largest stock in our study), all of which are also expected to experience range shifts resulting in decreased catch potential (Table 3). The stocks expected to experience a decrease catch potential from range shifts greater than 10% are mahi-mahi, pacific sardine, jumbo squid, black tip shark, red snapper, pelagic red crab, Spanish mackerel, pacific abalone, and snook (Table 3). Climate effects alone (independent differences in management scenarios) are projected to have the strongest negative effects on potential catch for mahi-mahi, pacific sardine, pelagic red crab, jumbo squid, and black tip shark (decreases in mean annual catch greater than 15%) (Fig 2).

However, despite the overall expected negative effects of climate change on Mexico’s fisheries, our results suggest that there is much to be gained through improved management. This result is driven by current unsustainable harvest rates in many of Mexico’s fisheries (see current fishing mortality rates (*F/F*_*MSY*_ values) in S1 Table). For the fisheries examined in this study, the expected benefits from management improvements are much larger than the projected losses due to moderate climate change. In fact, economically optimal management under climate change is expected to result in overall better economic and conservation outcomes than those that would be achieved under status quo management without climate change. Economically optimal management, which prevents overfishing by reducing catch when the stock is depleted, proves to be an effective management approach for a future with or without climate change (Fig 3). Although the economically optimal policy examined in this study is not optimized for the anticipated environmental changes, it still substantially outperforms the status quo policy in terms of profits and biomass. This is encouraging, since an adaptation strategy that relies on perfect responses to environmental changes requires certain knowledge of the pace and impacts of climate change, which is rarely available. This suggests that for these fisheries, flexible and adaptive management policies are worth implementing even if managers are unsure if and how climate change will affect fisheries.

Overall, our results suggest that the effects of climate change will vary among specific fisheries, fleets, and regions (Table 3, Figs 1–4). Surprisingly, despite having less ability to adapt to anticipated spatial shifts or local abundances, the resources caught by the artisanal fleet are expected to experience the lowest loss to net present value (NPV) directly from climate change. By contrast, species caught by the industrial fleet are expected to experience the greatest economic loss from climate change even though it has the greatest capacity for governance adaptation (Fig 5, Opt with CC compared to Opt no CC). Importantly, the majority of the volume caught by the industrial fleet–except for Pacific hake–were parameterized as having low adaptability to changes in catchability, because these species are expected to have larger range shifts beyond Mexico’s EEZ or fishable areas, suggesting that even comparatively adaptable fleets can be greatly impacted by range shifts that result in shifts across political boundaries. Regionally, fisheries in the Gulf of California and the Mexican Pacific are forecast to be some of the most heavily impacted by climate change. Almost all fisheries in these regions, with the exception of the cannonball jellyfish, experience declines in their productivity and catch potential. Given the results of this study, fisheries in the Gulf of California and the Mexican Pacific should be high priorities for future research, management and resources. Future work could more closely examine the vulnerability of stocks, fleets, and regions that we have prioritized as a result of this study, as well the potential for improved management.

Climate change is already having an impact in marine food resources, exacerbating the effects of overcapitalization and overfishing. Therefore, key challenges we must overcome are understanding how management should be adapted in the face of climate change and what we can expect in terms of fisheries biological, social and economic performance from improved management versus status quo. Our results for the Mexican cases analyzed here indicate that the answer varies across stocks and fleets, underscoring our hypothesis that more regionally specific analyses can help with stock management diagnostics and reform. Importantly, few fishing nations around the globe have seriously considered or taken pro-active measures to mitigate the impacts of climate change on their fisheries [5,23]. In 2018, Mexico updated its 2012 legislation (general law) dealing with mitigation and adaptation to climate change. Mexico could implement sustainable fishing practices under the framework of a national strategy to reduce climate change impacts on fisheries. Despite such efforts, strategies have not been effectively implemented to date. A review of laws deriving from the general law for the most important fishing states (Baja California, Sinaloa, Sonora and Veracruz) reveals that there are no fisheries adaptation strategies included. This is also reflected in the scarce number of published articles related to climate change impacts on fisheries in Mexico. We believe that the approach presented in this study can be used to highlight which stocks and regions are vulnerable to climate change and should be subject to closer investigation via improved data collection and further analysis.

## Assumptions and caveats/limitations

Our approach for estimating impacts relies on published literature and expert opinions of potential effects of climate change on marine species in different regions. There is, in general, only scarce information available for Mexico regarding how climate change may affect exploited species. Therefore, our parameter values can be considered a first, crude approximation of climate change impacts in Mexican fisheries. Given the data limitations, we assume that the variation in *g* and *K* is linear while in reality, changes may be highly non-linear. We made this assumption following Kritzer et al. [47], and recognize that this relationship could be revisited with improved data. There are also several important assumptions that we made for this study. First, the Pella-Tomlinson model is a surplus production model that does not capture effects of age structure. Our approach also uses a single-species model and therefore does not account for interactions among species or within ecosystems, which are likely to be impacted by climate change in complex ways. Furthermore, this model is not spatially explicit, but some of the anticipated effects of spatial shifts due to climate change are incorporated in the parameterization of catchability and reduced *in situ* biomass available to fishers. The model also excludes random effects in the environment or market. While these assumptions are relevant for fishery policy design, our goal for this study is to provide an approach for estimating potential climate impacts on fisheries and gleaning general insights on the potential effects of climate change on a subset of Mexican fisheries under different management policies. We do not explicitly take into consideration the fact that some firms or groups of fishers might shift from one species that is currently available to other stocks that shift into their fishing grounds, nor do we account for the fact that such species could be captured by fishers from other latitudes or by more technologically advanced fishers in the same site or region.

## Supporting information

S1 Text Implications of climate change on stocks included in the study. Explanations for the values displayed in Table 3.
(DOCX)

S2 Text Equation to calculate maximum sustainable yield. (DOCX)

S1 Table Input parameters for the stocks included in the study. (XLSX)

S2 Table Climate change parameterization for factors that affect growth rate *g*. (XLSX)

S3 Table Climate change parameterization for factors that affect carrying capacity *K*. (XLSX)

S4 Table Model outputs. (XLSX)

## References

[1] Brierley AS, Kingsford MJ. Impacts of Climate Change on Marine Organisms and Ecosystems. Curr Biol. 2009 7;19(14):R602–14. 10.1016/j.cub.2009.05.046 19640499

[2] Easterling W, Aggarwal P, Batima P, Brander K, Erda L, Howden M, et al Food, fibre and forest products.:42.

[3] Hoegh-Guldberg O, Bruno JF. The Impact of Climate Change on the World’s Marine Ecosystems. Science. 2010 6 18;328(5985):1523–8. 10.1126/science.1189930 20558709

[4] Rice JC, Garcia SM. Fisheries, food security, climate change, and biodiversity: characteristics of the sector and perspectives on emerging issues. ICES J Mar Sci. 2011 7 1;68(6):1343–53.

[5] Allison EH, Perry AL, Badjeck M-C, Adger WN, Brown K, Conway D, et al Vulnerability of national economies to the impacts of climate change on fisheries. Fish Fish. 2009;10(2):173–96.

[6] Brander KM. Global fish production and climate change. [cited 2019 Mar 18]; Available from: https://www.pnas.org/content/104/50/19709 10.1073/pnas.0702059104 PMC2148362 18077405

[7] Cheung WWL, Lam VWY, Sarmiento JL, Kearney K, Watson R, Zeller D, et al Large-scale redistribution of maximum fisheries catch potential in the global ocean under climate change. Glob Change Biol. 2010;16(1):24–35.

[8] Daw T, Adger WN, Brown K, Badjeck M-C. Climate change and capture fisheries: potential impacts, adaptation and mitigation.:44.

[9] Möllmann C, Diekmann R. Marine Ecosystem Regime Shifts Induced by Climate and Overfishing In: Advances in Ecological Research [Internet]. Elsevier; 2012 [cited 2019 Mar 18]. p. 303–47. Available from: https://linkinghub.elsevier.com/retrieve/pii/B9780123983152000041

[10] Rose GA. Reconciling overfishing and climate change with stock dynamics of Atlantic cod (*Gadus morhua*) over 500 years. Can J Fish Aquat Sci. 2004 9;61(9):1553–7.

[11] Rijnsdorp AD, Peck MA, Engelhard GH, Mollmann C, Pinnegar JK. Resolving the effect of climate change on fish populations. ICES J Mar Sci. 2009 8 1;66(7):1570–83.

[12] Barange M, Ian R. Perry. Physical and ecological impacts of climate change relevant to marine and inland capture fisheries and aquaculture. In: Climate change implications for fisheries and aquaculture. FAO; 2009.

[13] Rose G. On distributional responses of North Atlantic fish to climate change. ICES J Mar Sci. 2005 10;62(7):1360–74.

[14] Cheung WWL, Lam VWY, Sarmiento JL, Kearney K, Watson R, Pauly D. Projecting global marine biodiversity impacts under climate change scenarios. Fish Fish. 2009;10(3):235–51.

[15] Payne MR, Barange M, Cheung WWL, MacKenzie BR, Batchelder HP, Cormon X, et al Uncertainties in projecting climate-change impacts in marine ecosystems. ICES J Mar Sci J Cons. 2016 5;73(5):1272–82.

[16] Pinsky ML, Worm B, Fogarty MJ, Sarmiento JL, Levin SA. Marine Taxa Track Local Climate Velocities. Science. 2013 9 13;341(6151):1239–42. 10.1126/science.1239352 24031017

[17] Gaines SD, Costello C, Owashi B, Mangin T, Bone J, Molinos JG, et al Improved fisheries management could offset many negative effects of climate change. Sci Adv. 2018 8;4(8):eaao1378 10.1126/sciadv.aao1378 30167455 PMC6114984

[18] Christopher M. Free, Thornson JT, Pinsky ML, Oken KL, Wiednmann J, Jensen OP. Impacts of historical warming on marine fisheries production. Science. 2019;363:979–83. 10.1126/science.aau1758 30819962

[19] Perry AL, Low PJ, Ellis JR, Reynolds JD. Climate change and distribution shifts in marine fishes. science. 2005;308(5730):1912–1915. 10.1126/science.1111322 15890845

[20] Booth DJ, Feary D, Kobayashi D, Luiz O, Nakamura Y. Tropical Marine Fishes and Fisheries and Climate Change In: Phillips BF, Pérez-Ramírez M, editors. Climate Change Impacts on Fisheries and Aquaculture [Internet]. Chichester, UK: John Wiley & Sons, Ltd; 2017 [cited 2019 Jun 13]. p. 875–96. Available from: http://doi.wiley.com/10.1002/9781119154051.ch26

[21] García Molinos J, Halpern BS, Schoeman DS, Brown CJ, Kiessling W, Moore PJ, et al Climate velocity and the future global redistribution of marine biodiversity. Nat Clim Change. 2016 1;6(1):83–8.

[22] Barange M, Bahri T, Beveridge M, Cochrane K, Funge-Smith S, Poulain F. Impacts of climate change on fisheries and aquaculture: synthesis of current knowledge, adaptation and mitigation options. 2018.

[23] McIlgorm A, Hanna S, Knapp G, Le Floc’H P, Millerd F, Pan M. How will climate change alter fishery governanceʔ Insights from seven international case studies. Mar Policy. 2010 1;34(1):170–7.

[24] Costello C, Ovando D, Hilborn R, Gaines SD, Deschenes O, Lester SE. Status and Solutions for the World’s Unassessed Fisheries. Science. 2012 10 26;338(6106):517–20. 10.1126/science.1223389 23019613

[25] FAO, editor. The state of world fisheries and aquaculture—Meeting the sustainable development goals. Rome; 2018. 210 p.

[26] Oceana. Auditoría Pesquera: Pescando a ciegas [Internet]. Oceana; 2019 Jun [cited 2019 Jun 13]. Available from: https://auditoriapesquera.mx/wp-content/uploads/2019/06/OCEANA_Reporte_Auditoria-pesquera_web..pdf

[27] Costello C, Ovando D, Clavelle T, Strauss CK, Hilborn R, Melnychuk MC, et al Global fishery prospects under contrasting management regimes. Proc Natl Acad Sci. 2016 5 3;113(18):5125–9. 10.1073/pnas.1520420113 27035953 PMC4983844

[28] Pella JJ, Tomlinson PK. A generalized stock production model. Inter-American Tropical Tuna Commission Bulletin. 1969;13(3):416–97.

[29] Yoshimoto SS, Clarke RP. Comparing Dynamic Versions of the Schaefer and Fox Production Models and Their Application to Lobster Fisheries. Can J Fish Aquat Sci. 1993 1;50(1):181–9.

[30] Mangin T, Cisneros-Mata MÁ, Bone J, Costello C, Gaines SD, McDonald G, et al The cost of management delay: The case for reforming Mexican fisheries sooner rather than later. Mar Policy. 2018 2 1;88:1–10.

[31] Cisneros-Mata MÁ. Some guidelines for a reform in Mexican fisheries. 2016;15.

[32] Blanchard Julia L., Jennings Simon, Holmes Robert, Harle James, Merino Gorka, Allen J. Icarus, et al. Potential consequences of climate change for primary production and fish production in large marine ecosystems. Philos Trans R Soc B Biol Sci. 2012 11 5;367(1605):2979–89. 10.1098/rstb.2012.0231 PMC3479740 23007086

[33] Ji R, Edwards M, Mackas DL, Runge JA, Thomas AC. Marine plankton phenology and life history in a changing climate: current research and future directions. J Plankton Res. 2010 10 1;32(10):1355–68. 10.1093/plankt/fbq062 20824042 PMC2933132

[34] Ganster P, Arizpe C, Ivanova A. Los Cabos: Prospectiva de un Paraíso Natural y Turístico. San Diego State University Press; 2012.

[35] Saldívar-Lucio R, Salvadeo C, Del Monte-Luna P, Arreguín-Sánchez F, Villalobos H, Lluch-Belda D, et al Patrones históricos y escenarios térmicos futuros en mares mexicanos. Rev Biol Mar Oceanogr. 2015 8;50(2):331–45.

[36] Lenton A, Matear RJ, Mongin M. Effects of Climate Change on Ocean Acidification Relevant to the Pacific Islands.:12.

[37] Tzanatos E, Raitsos DE, Triantafyllou G, Somarakis S, Tsonis AA. Indications of a climate effect on Mediterranean fisheries. Clim Change. 2014 1;122(1–2):41–54.

[38] Weatherdon LV, Magnan AK, Rogers AD, Sumaila UR, Cheung WWL. Observed and Projected Impacts of Climate Change on Marine Fisheries, Aquaculture, Coastal Tourism, and Human Health: An Update. Front Mar Sci [Internet]. 2016 [cited 2019 Mar 18];3 Available from: https://www.frontiersin.org/articles/10.3389/fmars.2016.00048/full

[39] Travers-Trolet M, Shin Y-J, Shannon LJ, Moloney CL, Field JG. Combined Fishing and Climate Forcing in the Southern Benguela Upwelling Ecosystem: An End-to-End Modelling Approach Reveals Dampened Effects. Álvarez I, editor. PLoS ONE. 2014 4 7;9(4):e94286 10.1371/journal.pone.0094286 24710351 PMC3978043

[40] Holbrook NJ, Johnson JE. Climate change impacts and adaptation of commercial marine fisheries in Australia: a review of the science. Clim Change. 2014 6 1;124(4):703–15.

[41] Bakun A, Black BA, Bograd SJ, García-Reyes M, Miller AJ, Rykaczewski RR, et al Anticipated Effects of Climate Change on Coastal Upwelling Ecosystems. Curr Clim Change Rep. 2015 6;1(2):85–93.

[42] Hare JA, Morrison WE, Nelson MW, Stachura MM, Teeters EJ, Griffis RB, et al A Vulnerability Assessment of Fish and Invertebrates to Climate Change on the Northeast U.S. Continental Shelf. Hiddink JG, editor. PLOS ONE. 2016 2 3;11(2):e0146756 10.1371/journal.pone.0146756 26839967 PMC4739546

[43] Daw TM, Cinner JE, McClanahan TR, Brown K, Stead SM, Graham NAJ, et al To Fish or Not to Fish: Factors at Multiple Scales Affecting Artisanal Fishers’ Readiness to Exit a Declining Fishery. Clifton J, editor. PLoS ONE. 2012 2 10;7(2):e31460 10.1371/journal.pone.0031460 22348090 PMC3277441

[44] Arroyo Martínez A, Manzanilla Naim S, Zavala Hidalgo J. Vulnerability to climate change of marine and coastal fisheries in México. Atmosfera. 2011;24(1):103–23.

[45] Robinson CJ, Gómez-Gutiérrez J, Markaida U, Gilly WF. Prolonged decline of jumbo squid (Dosidicus gigas) landings in the Gulf of California is associated with chronically low wind stress and decreased chlorophyll a after El Niño 2009–2010. Fish Res. 2016 1 1;173:128–38.

[46] Litz MNC, Phillips AJ, Brodeur RD, Emmett RL. SEASONAL OCCURRENCES OF HUMBOLDT SQUID (DOSIDICUS GIGAS) IN THE NORTHERN CALIFORNIA CURRENT SYSTEM. 2011;52:12.

[47] Kritzer JP, Costello C, Mangin T, Smith SL. Responsive harvest control rules provide inherent resilience to adverse effects of climate change and scientific uncertainty. Prellezo R, editor. ICES J Mar Sci [Internet]. 2019 4 1 [cited 2019 Jun 13]; Available from: https://academic.oup.com/icesjms/advance-article/doi/10.1093/icesjms/fsz038/5425355

