# Supplementary material for: Fisheries governance in the face of climate change: Assessment of policy reform implications for Mexican fisheries
Source: PLoS One. 2019 Oct 2;14(10):e0222317. doi: 10.1371/journal.pone.0222317 (PMC6774473; doi:10.1371/journal.pone.0222317)
Supplement: S1 Text — Explanations for the values displayed in Table 3. (DOCX) [file pone.0222317.s001.docx]

**S1 Text. Implications of climate change on stocks in study.** Explanations for the values displayed in Table 3.

**Black murex snail (*Hexaplex nigritus*)**

Temperature: Annual sea surface temperature in the northern Gulf of California ranges from 17°C to 30°C; hence forcing is not likely [1].

Acidification: We use a low negative effect because while some acidification is expected in the Gulf of California, strong acidification is not predicted [2].

Disease outbreaks: Outbreaks not likely, as there is not much human or industrial development northern Gulf of California.

Sea level rise: We use a medium positive effect, as sea level rise is likely to increase habitat volume.

Fresh water inflow: We did not find evidence for an expected effect.

Migration: Not likely to be affected due to fidelity to reproduction sites [3].

Catchability: Not likely to be affected due to gregarious behavior [3].

Governance: We use a low negative effect because of illegal fishing [4].

**Brown swimming crab (*Callinectes bellicosus*)**

Temperature: This species inhabits all of the Mexican Pacific ]5], and thus is not expected to be impacted by changes in sea surface temperature.

Acidification: Might slightly reduce crab´s ability to predate ]6]. Therefore, we use a low negative effect.

Disease outbreaks: We use a low negative effect because juveniles grow in coastal lagoons, which are subject to diseases, e.g. from shrimp cultivation ]7].

Sea level rise: Expected to slightly increase habitat volume and refugia for juveniles in coastal lagoons. Therefore, we use a medium positive effect.

Freshwater inflow: We use a medium positive effect because freshwater inflow is expected to improve habitat quality and size.

Migration: We use a low negative effect because of anticipated larval drift and range shifts for adults ]8].

Catchability: No effect is expected because the fishing communities innovate gear.

Governance: We use a low negative effect because illegal fishing slightly impairs sustainability in this fishery ]4].

**Cannonball jellyfish (*Stomolophus* spp.)**

Temperature: Linked with population increases across the globe, and therefore we use a medium positive effect [9].

Acidification: Expected to negatively affect larvae [10]. Therefore, we use a low negative effect.

Disease outbreaks: We did not find evidence for an expected effect.

Sea level rise: We use a medium positive effect because sea level rise is expected to increase habitat size in nursery areas [11].

Freshwater inflow: We did not find evidence for an expected effect.

Migration: We use a low negative effect, as moderate shifts to northern latitudes are possible.

Catchability: No effect is expected as fishers promptly innovate.

Governance: We use a low negative effect because of the almost de facto open access fishery, which is likely to affect sustainable fishing [12].

**Chocolate clam (*Megapteria squalida*)**

Temperature: No effect expected [13].

Acidification: We use a low negative impact, as most clams are expected to experience a negative effect [14].

Disease outbreaks: Since this area is relatively unpopulated and therefore lacks much development, climate change is not considered to affect disease outbreaks.

Sea level rise: Positive habitat augmentation expected, and hence we use a medium positive effect.

Freshwater inflow: Because expected decreases in salinity would affect adults, we use a moderate negative effect.

Migration: We did not find evidence for an expected effect.

Catchability: Not expected to be altered as fishers innovate constantly.

Governance: We use a low negative effect is considered because of presence of illegal fishing [4].

**Geoduck (*Panopea globose*)**

Temperature: We use a low positive effect because increased sea surface temperature positively affects recruitment [15].

Acidification: We use a low negative effect is because acidification is expected to negatively affect recruitment [14].

Disease outbreaks: We did not find evidence for an expected effect.

Sea level rise: We use a medium positive effect because sea level rise would increase habitat size.

Freshwater inflow: We use a low negative effect because anticipated freshwater inflow may reduce recruitment [15].

Migration: We did not find evidence for an expected effect.

Catchability: No effect is expected due to highly specialized fishing.

Governance: We use a low negative effect because of illegal fishing [4].

**Gulf corvina (*Cynoscion othonopterus*)**

Temperature: The stock has a large range of temperature habitat; therefore, we assume no effect.

Acidification: We use a low negative impact because of anticipated effects on larval survival [16–18].

Disease outbreaks: We did not find evidence for an expected effect.

Sea level rise: Habitat increase may result in higher survival and recruitment. Therefore, we use a medium positive effect.

Fresh water inflow: Anticipated to lead to decreased larval survival. Therefore, we use a medium negative effect.

Migration: This species is endemic, and thus no expected effects.

Catchability: Fishers enclose nets over spawning aggregations, and thus no effect expected.

Governance: No effect expected because the fishery is well organized [4].

**Lion-paw clam (*Lyropecten subnodosus*)**

Temperature: No effect is expected due to ample distribution [19].

Acidification: We use a medium negative effect as there is ample evidence suggesting all mollusks will be negatively affected by ocean acidification [20,21].

Disease outbreaks: We did not find evidence for an expected effect.

Sea level rise: We use a medium positive effect as sea level rise is expected to increase habitat size.

Fresh water inflow: We did not find evidence for an expected effect.

Migration: We did not find evidence for an expected effect.

Catchability: No effects expected.

Governance: We use a low negative effect because of illegal fishing [4].

**Pacific abalone (*Haliotis fulgens*)**

Temperature: We use a medium negative effect because of decreased food availability [22].

Acidification: We use a medium negative effect because of reduced survival and recruitment [20,21].

Disease outbreaks: We use a high negative effect because evidence suggests that abalones may be strongly impacted by increases in disease outbreaks [23].

Sea level rise: We did not find evidence for an expected effect.

Fresh water inflow: We use a medium negative effect because of decreased recruitment.

Migration: We use a medium negative effect because of anticipated larval drift in currents and North-bound shifting adults [8].

Catchability: No effect is expected due to highly specialized fishing.

Governance: No effect is expected because fishers are highly organized [4].

**Penshell scallop (*Atrina tuberculosa*)**

Temperature: No effect is expected due to active ocean circulation in the study area [24].

Acidification: We use a low negative effect is considered [14,25].

Disease outbreaks: We did not find evidence for an expected effect.

Sea level rise: We use a low positive effect because of anticipated increases in habitat.

Fresh water inflow: No effect expected in the dry study area.

Migration: We did not find evidence for an expected effect.

Catchability: No effect is expected due to highly specialized fishing [4].

Governance: We use a medium negative effect because of illegal fishing [4].

**Queen conch (*Strombus gigas*)**

Temperature: We did not find evidence for an expected effect.

Acidification: We use a low negative effect [20,21,26].

Disease outbreaks: We did not find evidence for an expected effect.

Sea level rise: We use a low positive effect because of anticipated expansions in habitat.

Fresh water inflow: We did not find evidence for an expected effect.

Migration: We did not find evidence for an expected effect.

Catchability: No effect is expected because the fishery is specialized.

Governance: We use a low negative effect because of illegal fishing [4].

**Red snapper (*Lutjanus peru*)**

Temperature: We use a low negative impact because of expected negative effects on recruitment [27].

Acidification: We use a low negative impact because of anticipated effects on larval survival [16–18].

Disease outbreaks: We did not find evidence for an expected effect.

Sea level rise: We use a low positive effect because of anticipated increases in habitat for larval stages.

Fresh water inflow: We did not find evidence for an expected effect.

Migration: We use a medium negative effect because of projected range shifts [8,28].

Catchability: We use a low negative effect because of poor specialization within the fishery [4].

Governance: We use a medium negative effect because of illegal fishing [4].

**Sea cucumber (*Isostichopus fuscus*)**

Temperature: We use a low positive effect [29].

Acidification: We use a low negative effect [30].

Disease outbreaks: We did not find evidence for an expected effect.

Sea level rise: We use a low positive effect because of anticipated increases in habitat.

Fresh water inflow: We did not find evidence for an expected effect.

Migration: We did not find evidence for an expected effect.

Catchability: No effect is expected because the fishery is specialized.

Governance: We use a medium effect because of illegal fishing [4].

**Snook (*Centropomus robalito*)**

Temperature: No expected effect for this subtropical species.

Acidification: We use a low negative impact because of anticipated effects on larval survival [16–18].

Disease outbreaks: We did not find evidence for an expected effect.

Sea level rise: We use a medium positive effect because of anticipated increases in habitat.

Fresh water inflow: We use a medium positive effect for this estuarine species [31].

Migration: We use a medium negative effect, as these euryhaline stocks could shift to adjacent estuaries [8,32].

Catchability: We did not find evidence for an expected effect.

Governance: We use a medium negative effect because of illegal fishing [4].

**Spanish mackerel (*Scomberomorus* spp*.*)**

Temperature: We did not find evidence for an expected effect.

Acidification: We use a low negative impact because of anticipated effects on larval survival [16–18].

Disease outbreaks: We did not find evidence for an expected effect.

Sea level rise: We did not find evidence for an expected effect.

Fresh water inflow: We did not find evidence for an expected effect.

Migration: We use a low negative effect [33].

Catchability: We did not find evidence for an expected effect.

Governance: We use a medium negative effect because of illegal fishing [4].

**Spiny lobster (*Panulirus interruptus*)**

Temperature: We do not include an effect due to mixed information [34].

Acidification: We use a low negative effect because of anticipated impaired foraging [6].

Disease outbreaks: We did not find evidence for an expected effect.

Sea level rise: We use a low positive effect because of anticipated increases in habitat.

Fresh water inflow: We did not find evidence for an expected effect.

Migration: We use a medium effect because of predicted larval drift [35].

Catchability: No effects considered for this specialized fishery.

Governance: No effects is expected because the relevant fishing communities are well organized [4].

**Triggerfish (*Ballistes polylepis*)**

Temperature: No impact considered because this species is widely distributed species.

Acidification: We use a low negative impact because of anticipated effects on larval survival [16–18].

Disease outbreaks: We did not find evidence for an expected effect.

Sea level rise: We did not find evidence for an expected effect.

Fresh water inflow: We did not find evidence for an expected effect.

Migration: No effect is expected due to ample distribution [36].

Catchability: No effect is expected due to a variety of fishing methods (nets, pots, hook and line).

Governance: We use a medium negative effect because of open access fishing pressure [4].

**Pacific hake (*Merluccius productus*)**

Temperature: Not considered to affect this largely mesopelagic resource [37].

Acidification: We use a low negative impact because of anticipated effects on larval survival [16–18].

Disease outbreaks: We did not find evidence for an expected effect.

Sea level rise: We did not find evidence for an expected effect.

Fresh water inflow: We did not find evidence for an expected effect.

Migration: We did not find evidence for an expected effect.

Catchability: No impact is expected for this specialized fishery.

Governance: We use a low negative effect because of unreported catch [4].

**Pacific sardine (*Sardinops sagax*)**

Temperature: We use a medium negative effect because of expected larval mortality [38,39].

Acidification: We use a low negative impact because of anticipated effects on larval survival [16–18].

Disease outbreaks: We did not find evidence for an expected effect.

Sea level rise: We did not find evidence for an expected effect.

Fresh water inflow: We did not find evidence for an expected effect.

Migration: We use a high negative impact because of anticipated vertical and horizontal shifts.

Catchability: We use a medium negative impact because of impaired accessibility.

Governance: No effect is expected because the relevant fishing industry is highly organized [4].

**Pelagic red crab (*Pleuroncodes planipes*)**

Temperature: We use a medium negative effect because of anticipated impact on survival [40,41].

Acidification: We use a medium negative effect because of the anticipated impact on adults and larvae [6].

Disease outbreaks: We did not find evidence for an expected effect.

Sea level rise: We did not find evidence for an expected effect.

Fresh water inflow: We did not find evidence for an expected effect.

Migration: We use a medium negative effect because of anticipated horizontal shifts [40].

Catchability: We use a medium negative effect because of reduced vulnerability.

Governance: No impact is expected because the relevant fleet is highly organized [4].

**Yellowfin tuna (*Thunnus albacares*)**

Temperature: No effects are considered for this highly migratory species.

Acidification: We use a low negative impact because of anticipated effects on larval survival [16–18].

Disease outbreaks: We did not find evidence for an expected effect.

Sea level rise: We did not find evidence for an expected effect.

Fresh water inflow: We did not find evidence for an expected effect.

Migration: We use a low negative effect because of latitudinal shifts [41,42].

Catchability: We did not find evidence for an expected effect.

Governance: No impact is expected because the relevant fleet is highly organized [4].

**Black tip shark (*Carcharhinus limbatus*)**

Temperature: We did not find evidence for an expected effect.

Acidification: We did not find evidence for an expected effect.

Disease outbreaks: We did not find evidence for an expected effect.

Sea level rise: We did not find evidence for an expected effect.

Fresh water inflow: We did not find evidence for an expected effect.

Migration: We use a low negative effect because of expected horizontal shifts [43,44].

Catchability: We use a medium negative effect because of reduced vulnerability.

Governance: We used a medium negative effect because of illegal fishing [4].

**Blue shrimp (*Litopenaeus stylirostris*)**

Temperature: No effect is expected for this tolerant species which inhabits coastal and inshore waters [45].

Acidification: We use a low negative effect [6].

Disease outbreaks: We use a low negative effect [46,47].

Sea level rise: We did not find evidence for an expected effect.

Fresh water inflow: We use a low positive effect [48].

Migration: No effect is expected due to its wide distribution.

Catchability: No effect is expected because of different gear and fleets participating in the fishery.

Governance: We use a low negative effect because of illegal fishing [4].

**Jumbo squid (*Dosidicus gigas*)**

Temperature: We use a low negative impact because of decreased food intake [49].

Acidification: We use a low negative effect because of anticipated impacts to metabolism [50].

Disease outbreaks: We did not find evidence for an expected effect.

Sea level rise: We did not find evidence for an expected effect.

Fresh water inflow: No effect is expected for this abundant species in the Eastern Tropical Pacific [51].

Migration: We use a high negative effect because of latitudinal shifts [51,52].

Catchability: We use a low negative effect because of temperature-related distribution and low specialization of fishery [4,53].

Governance: We use a low negative effect because of illegal fishing [4].

**Mahi-mahi (*Coryphaena* spp*.*)**

Temperature: No effect is expected for this wide-ranging species ]54,55].

Acidification: We use a low negative effect because of anticipated effects on larval survival ]16–18].

Disease outbreaks: We did not find evidence for an expected effect.

Sea level rise: We did not find evidence for an expected effect.

Fresh water inflow: We did not find evidence for an expected effect.

Migration: We use a medium negative effect because of anticipated shifts in distribution ]8,54].

Catchability: We use a medium negative effect because of expected range shifts.

Governance: We use a medium negative effect because of illegal fishing ]4].

**Red grouper (*Epinephelus morio*)**

Temperature: We use a low negative effect [56].

Acidification: We use a low negative impact because of anticipated effects on larval survival [16–18].

Disease outbreaks: We did not find evidence for an expected effect.

Sea level rise: We did not find evidence for an expected effect.

Fresh water inflow: We did not find evidence for an expected effect.

Migration: We use a low negative effect considered anticipated range shifts [8].

Catchability: We did not find evidence for an expected effect.

Governance: We use a low negative effect because of illegal fishing [4].

**References**

1. Rodríguez-Félix D, Cisneros-Mata MÁ, Aragón-Noriega EA. Variability of size at maturity of the warrior swimming crab, Callinectes bellicosus (Stimpson, 1859) (Brachyura, Portunidae), along a latitudinal gradient in the Gulf of California. Crustaceana. 2015 Jan 1;88(9):979–89.

2. Páez-Osuna F, Sanchez-Cabeza JA, Ruiz-Fernández AC, Alonso-Rodríguez R, Piñón-Gimate A, Cardoso-Mohedano JG, et al. Environmental status of the Gulf of California: A review of responses to climate change and climate variability. Earth-Science Reviews. 2016 Nov 1;162:253–68.

3. Cudney-Bueno R, Prescott R, Hinojosa-Huerta O. The Black Murex Snail, Hexaplex Nigritus (Mollusca, Muricidae), in the Gulf of California, Mexico: I. Reproductive Ecology and Breeding Aggregations [Internet]. 2008 [cited 2019 Aug 22]. Available from: https://www.ingentaconnect.com/content/umrsmas/bullmar/2008/00000083/00000002/art00001

4. Cisneros-Mata MÁ. Some guidelines for a reform in Mexican fisheries. 2016;15.

5. Hendrickx M. Cangrejos. In: Guía FAO para la identificación de especies para los fines de la pesca Pacífico Centro-oriental. FAO, Rome; 1995. p. 565–636.

6. Dodd Luke F., Grabowski Jonathan H., Piehler Michael F., Westfield Isaac, Ries Justin B. Ocean acidification impairs crab foraging behaviour. Proceedings of the Royal Society B: Biological Sciences. 2015 Jul 7;282(1810):20150333.

7. Cisneros-Mata M, Ramírez-Félix E, Garcia-Borbon J, Castañeda-Fernandez de Lara V, Labastida-Che A, Gómez-Rojo C, et al. Pesca de jaiba en el litoral del Pacífico mexicano. Instituto Nacional de Pesca, SAGARPA, Mexico City; 2014.

8. Cheung WWL, Lam VWY, Pauly D. Modelling present and climate-shifted distribution of marine fishes and invertebrates. 2008 [cited 2019 Aug 22]; Available from: https://open.library.ubc.ca/cIRcle/collections/facultyresearchandpublications/52383/items/1.0074754

9. Richardson AJ, Bakun A, Hays GC, Gibbons MJ. The jellyfish joyride: causes, consequences and management responses to a more gelatinous future. Trends in Ecology & Evolution. 2009 Jun 1;24(6):312–22.

10. Dong Z, Sun T. Combined effects of ocean acidification and temperature on planula larvae of the moon jellyfish Aurelia coerulea. Marine Environmental Research. 2018 Aug 1;139:144–50.

11. Carvalho-Saucedo L, López-Martínez J, García-Domínguez F, Rodríguez-Jaramillo C, Padilla-Serrato J. Reproductive biology of the cannonball jellyfish Stomolophus meleagris in las guasimas Lagoon, Sonora, Mexico. Hidrobiologica. 2011 Apr 1;77–88.

12. Cruz-Colín M, Cisneros-Mata M, Montaño-Moctezuma G. Análisis de actores de la pesquería de medusa en Guaymas, Sonora. Región y Sociedad. 2019;31.

13. Xiao B, Li E, Du Z, Jiang R, Chen L, Yu N. Effects of temperature and salinity on metabolic rate of the Asiatic clam Corbicula fluminea (Müller, 1774). SpringerPlus. 2014 Aug 22;3(1):455.

14. Mangi SC, Lee J, Pinnegar JK, Law RJ, Tyllianakis E, Birchenough SNR. The economic impacts of ocean acidification on shellfish fisheries and aquaculture in the United Kingdom. Environmental Science & Policy. 2018 Aug 1;86:95–105.

15. Valero JL, Hand C. GEODUCK (PANOPEA ABRUPTA) RECRUITMENT IN THE PACIFIC NORTHWEST: LONG-TERM CHANGES IN RELATION TO CLIMATE. 2004;45:7.

16. Bignami S, Sponaugle S, Cowen RK. Effects of ocean acidification on the larvae of a high-value pelagic fisheries species, mahi-mahi Coryphaena hippurus. Aquatic Biology. 2014 Oct 29;21(3):249–60.

17. Pimentel M, Pegado M, Repolho T, Rosa R. Impact of ocean acidification in the metabolism and swimming behavior of the dolphinfish (Coryphaena hippurus) early larvae. Mar Biol. 2014 Mar 1;161(3):725–9.

18. Dell’Apa A, Carney K, Davenport TM, Carle MV. Potential medium-term impacts of climate change on tuna and billfish in the Gulf of Mexico: A qualitative framework for management and conservation. Marine Environmental Research. 2018 Oct 1;141:1–11.

19. Taylor MH, Koch V, Wolff M, Sínsel F. Evaluation of different shallow water culture methods for the scallop Nodipecten subnodosus using biologic and economic modeling. Aquaculture. 2006 Apr 28;254(1):301–16.

20. Guo X, Huang M, Pu F, You W, Ke C. Effects of ocean acidification caused by rising CO2 on the early development of three mollusks. Aquatic Biology. 2015 Feb 10;23(2):147–57.

21. Li J, Mao Y, Jiang Z, Zhang J, Fang J, Bian D. The detrimental effects of CO2-driven chronic acidification on juvenile Pacific abalone (Haliotis discus hannai). Hydrobiologia. 2018 Mar 1;809(1):297–308.

22. Vilchis LI, Tegner MJ, Moore JD, Friedman CS, Riser KL, Robbins TT, et al. Ocean Warming Effects on Growth, Reproduction, and Survivorship of Southern California Abalone. Ecological Applications. 2005;15(2):469–80.

23. Moore JD, Juhasz CI, Robbins TT, Vilchis LI. Green abalone, Haliotis fulgens infected with the agent of withering syndrome do not express disease signs under a temperature regime permissive for red abalone, Haliotis rufescens. Mar Biol. 2009 Oct 1;156(11):2325–30.

24. Alvarez-Borrego S, Lara-Lara JR. The Physical Environment and Primary Productivity of the Gulf of California: Chapter 26: Part V. Physical Oceanography, Primary Productivity, Sedimentology. 1991;114:555–67.

25. Rheuban JE, Doney SC, Cooley SR, Hart DR. Projected impacts of future climate change, ocean acidification, and management on the US Atlantic sea scallop (Placopecten magellanicus) fishery. PLOS ONE. 2018 Sep 21;13(9):e0203536.

26. Aranda DA, Manzano NB. Effects of near-future-predicted ocean temperatures on early development and calcification of the queen conch Strombus gigas. Aquacult Int. 2017 Oct 1;25(5):1869–81.

27. Martínez Arroyo A, Manzanilla Naim S, Zavala Hidalgo J. Vulnerability to climate change of marine and coastal fisheries in México. Atmósfera. 2011;41(1):103–23.

28. Hare JA, Wuenschel MJ, Kimball ME. Projecting Range Limits with Coupled Thermal Tolerance - Climate Change Models: An Example Based on Gray Snapper (Lutjanus griseus) along the U.S. East Coast. PLOS ONE. 2012 Dec 20;7(12):e52294.

29. Defeo O, Castrejón M, Ortega L, Kuhn A, Gutiérrez N, Castilla JC. Impacts of Climate Variability on Latin American Small-scale Fisheries. Ecology and Society [Internet]. 2013 Nov 8 [cited 2019 Aug 22];18(4). Available from: https://www.ecologyandsociety.org/vol18/iss4/art30/

30. Dupont S, Ortega-Martínez O, Thorndyke M. Impact of near-future ocean acidification on echinoderms. Ecotoxicology. 2010 Mar;19(3):449–62.

31. Boucek RE, Heithaus MR, Santos R, Stevens P, Rehage JS. Can animal habitat use patterns influence their vulnerability to extreme climate events? An estuarine sportfish case study. Global Change Biology. 2017;23(10):4045–57.

32. Andrade H, Santos J, Taylor R. Life-history traits of the common snook Centropomus undecimalis in a Caribbean estuary and large-scale biogeographic patterns relevant to management. Journal of Fish Biology. 2013;82(6):1951–74.

33. Vaidyanathan G. Inner Workings: Climate change complicates fisheries modeling and management. PNAS. 2017 Aug 8;114(32):8435–7.

34. Tlusty M, Metzler A, Malkin E, Goldstein J, Koneval M. Microecological Impacts of Global Warming on Crustaceans—Temperature Induced Shifts in the Release of Larvae from American Lobster, Homarus americanus, Females. shre. 2008 Apr;27(2):443–8.

35. Cetina‐Heredia P, Roughan M, Sebille E van, Feng M, Coleman MA. Strengthened currents override the effect of warming on lobster larval dispersal and survival. Global Change Biology. 2015;21(12):4377–86.

36. Allen G, Bauchot M, Bellwood D, Bianchi G, Bussing W, Caruso J. Peces óseos. In: Guía FAO para la identificación de especies para los fines de la pesca Pacífico Centro-oriental. Rome: FAO; 1995.

37. Alverson DL, Larkins HA. Status of knowledge of the Pacific hake resource. Calif Coop Oceanic Fish Invest Rep. 1969;13:24–31.

38. Sánchez-Velasco L, Valdez-Holguı́n JE, Shirasago B, Cisneros-Mata MA, Zarate A. Changes in the Spawning Environment of Sardinops caeruleus in the Gulf of California during El Niño 1997–1998. Estuarine, Coastal and Shelf Science. 2002 Feb 1;54(2):207–17.

39. Petatán-Ramírez D, Ojeda-Ruiz MÁ, Sánchez-Velasco L, Rivas D, Reyes-Bonilla H, Cruz-Piñón G, et al. Potential changes in the distribution of suitable habitat for Pacific sardine (Sardinops sagax) under climate change scenarios. Deep Sea Research Part II: Topical Studies in Oceanography. 2019 Aug 1;104632.

40. De Anda-Montañez JA, Martínez-Aguilar S, Balart EF, Zenteno-Savín T, Méndez-Rodríguez L, Amador-Silva E, et al. Spatio-temporal distribution and abundance patterns of red crab Pleuroncodes planipes related to ocean temperature from the Pacific coast of the Baja California Peninsula. Fish Sci. 2016 Jan 1;82(1):1–15.

41. Dell JT, Wilcox C, Matear RJ, Chamberlain MA, Hobday AJ. Potential impacts of climate change on the distribution of longline catches of yellowfin tuna (Thunnus albacares) in the Tasman sea. Deep Sea Research Part II: Topical Studies in Oceanography. 2015 Mar 1;113:235–45.

42. Monllor-Hurtado A, Pennino MG, Sanchez-Lizaso JL. Shift in tuna catches due to ocean warming. Clark TD, editor. PLOS ONE. 2017 Jun 7;12(6):e0178196.

43. Castro JI. Biology of the Blacktip Shark, Carcharhinus Limbatus, off the Southeastern United States [Internet]. 1996 [cited 2019 Aug 22]. Available from: https://www.ingentaconnect.com/content/umrsmas/bullmar/1996/00000059/00000003/art00005

44. Kajiura SM, Tellman SL. Quantification of Massive Seasonal Aggregations of Blacktip Sharks (Carcharhinus limbatus) in Southeast Florida. Patterson HM, editor. PLOS ONE. 2016 Mar 30;11(3):e0150911.

45. López-Martínez J, Rábago-Quiroz C, Nevárez-Martínez MO, García-Juárez AR, Rivera-Parra G, Chávez-Villalba J. Growth, reproduction, and size at first maturity of blue shrimp, Litopenaeus stylirostris (Stimpson, 1874) along the east coast of the Gulf of California, Mexico. Fisheries Research. 2005 Jan 1;71(1):93–102.

46. Morales‐Covarrubias MS, Nunan LM, Lightner DV, Mota‐Urbina JC, Garza‐Aguirre MC, Chávez‐Sánchez MC. Prevalence of Infectious Hypodermal and Hematopoietic NecrosisVirus (IHHNV) in Wild Adult Blue Shrimp Penaeus stylirostris from the Northern Gulf of California, Mexico. Journal of Aquatic Animal Health. 1999;11(3):296–301.

47. Islam MA, Islam MS, Wahab MA. Impacts of climate change on shrimp farming in the South-West coastal region of Bangladesh. 1. 2016 May 26;3(1):227–39.

48. Aragón-Noriega EA, Calderón-Aguilera LE. Does damming of the Colorado River affect the nursery area of blue shrimp Litopenaeus stylirostris (Decapoda: Penaeidae) in the Upper Gulf of California? Revista de Biología Tropical. 2000 Dec;48(4):867–71.

49. Rosa R, Seibel BA. Synergistic effects of climate-related variables suggest future physiological impairment in a top oceanic predator. PNAS. 2008 Dec 30;105(52):20776–80.

50. Seibel BA. Environmental Physiology of the Jumbo Squid, Dosidicus gigas (d’Orbigny, 1835) (Cephalopoda: Ommastrephidae): Implications for Changing Climate *. malb. 2015 Feb;33(1):161–73.

51. Field JC, Baltz K, Phillips AJ. RANGE EXPANSION AND TROPHIC INTERACTIONS OF THE JUMBO SQUID, DOSIDICUS GIGAS, IN THE CALIFORNIA CURRENT. 2007;48:16.

52. Zeidberg LD, Robison BH. Invasive range expansion by the Humboldt squid, Dosidicus gigas, in the eastern North Pacific. PNAS. 2007 Jul 31;104(31):12948–50.

53. Robinson CJ, Gómez-Gutiérrez J, de León DAS. Jumbo squid (Dosidicus gigas) landings in the Gulf of California related to remotely sensed SST and concentrations of chlorophyll a (1998–2012). Fisheries Research. 2013 Jan 1;137:97–103.

54. Norton JG. Apparent habitat extensions of dolphinfish (Coryphaena hippurus) in response to climate transients in the California Current. Scientia Marina. 1999 Dec 30;63(3–4):239–60.

55. Olson RJ, Galván-Magaña F. Food habits and consumption rates of common dolphinfish (Coryphaena hippurus) in the eastern Pacific Ocean. Fishery Bulletin. 2002;100:279–98.

56. Pratchett MS, Cameron DS, Donelson J, Evans L, Frisch AJ, Hobday AJ, et al. Effects of climate change on coral grouper (Plectropomus spp.) and possible adaptation options. Rev Fish Biol Fisheries. 2017 Jun 1;27(2):297–316.
