# Supplementary material for: Fisheries governance in the face of climate change: Assessment of policy reform implications for Mexican fisheries
Source: PLoS One. 2019 Oct 2;14(10):e0222317. doi: 10.1371/journal.pone.0222317 (PMC6774473; doi:10.1371/journal.pone.0222317)
Supplement: S2 Text — (DOCX) [file pone.0222317.s002.docx]

**S2 Text. Equation to calculate maximum sustainable yield.** This equation is a modified version of that found in Costello et al. to include time period *t* [1]. Maximum sustainable yield in time *t* depends on *g* and *K* in time *t.*

$${MSY}_{t}= \frac{g_{t}K_{t}}{{(\phi+1)}^{\frac{1}{\phi}}}$$

**Reference**

1. Costello C, Ovando D, Clavelle T, Strauss CK, Hilborn R, Melnychuk MC, et al. Global fishery prospects under contrasting management regimes. PNAS. 2016 May 3;113(18):5125–9.
